# Acute alcohol-related dysfunction as a predictor of employment status in a longitudinal study of working-age men in Izhevsk, Russia

**DOI:** 10.1111/add.12329

**Published:** 2013-09-13

**Authors:** Sarah Cook, Bianca L DeStavola, Lyudmila Saburova, David A Leon

**Affiliations:** London School of Hygiene & Tropical MedicineLondon, UK1; Izhevsk State Technical UniversityIzhevsk, Russia2

**Keywords:** Alcohol, alcohol-related dysfunction, drunkenness, employment, hangover Russia

## Abstract

**Aims:**

To investigate longitudinally the effect of alcohol consumption and related acute alcohol-related dysfunction on employment status.

**Design, setting and participants:**

A total of 1143 men aged 25–55 years in regular paid employment and resident in the city of Izhevsk, Russia were interviewed between 2003–06 and then re-interviewed (2008–09) and their employment status ascertained.

**Measurements:**

Exposures of interest were baseline alcohol intake (yearly total volume of ethanol consumed and non**-**beverage alcohols) and alcohol-related dysfunction, measured by a latent variable defined in terms of frequency of alcohol-related dysfunctional behaviours and by one or more episodes of *zapoi* (a period of continuous drunkenness lasting 2 or more days). The outcome of interest was whether or not men were still in regular paid employment at follow-up. The inter-relationship between these variables was investigated using structural equation modelling.

**Findings:**

Total volume of ethanol consumed had no substantive effect on future employment status; however, taking into account education and other socio-demographic factors, there was strong evidence that loss of regular paid employment at follow-up was influenced by non-beverage alcohol consumption [odds ratio = 2.30 for non-beverage drinkers compared with beverage-only drinkers, 95% confidence interval (CI) = 1.21, 4.40)], latent acute alcohol-related dysfunction (odds ratio = 1.50 per standard deviation increase in dysfunction score, 95% CI = 1.20, 1.88) and *zapoi* (odds ratio = 3.08, 95% CI = 1.71, 5.55). Acute alcohol-related dysfunction was an important mediator of the relationship between non-beverage alcohol use and employment status.

**Conclusions:**

Acute alcohol-related dysfunction is an important factor in determining whether men remain in employment and an important mediator of the effects of alcohol intake.

## INTRODUCTION

As well as having a negative impact on health [1], heavy alcohol consumption can also have a negative effect on an individual's welfare through its effects on work-place functioning and employment status [2–7]. Loss of employment itself can have many negative effects, including poverty, marginalization and adverse mental and physical health outcomes [8,9].

Pathways from alcohol consumption to unemployment may be through chronic effects on health which make it difficult or impossible to remain in work, but also through acute dysfunctional consequences of alcohol such as hangover, which can directly affect an individual's ability to function in the work-place. Whether or not alcohol consumption results in acute dysfunctional behaviour could be particularly important for any effect of alcohol use on employment status. Acute dysfunction may be an important mediator between alcohol intake and loss of employment, because even when consuming the same volume of ethanol individuals can differ in their vulnerability to dysfunction and therefore the impact of their drinking on their work life.

The majority of longitudinal studies investigating alcohol consumption as a predictor of employment have investigated only the effects of volume of ethanol consumed [2,3]. Only one study used a measure of acute dysfunction: Liira *et al*. found that self-reported drunkenness once a week or more predicted employment status in Scandinavian construction workers, but not forest workers [4]. However, it is unlikely that drunkenness once a week or more captured men's experience of acute dysfunction adequately. In addition, this study did not measure the amount of ethanol consumed, so could not investigate how volume of ethanol, acute dysfunction and employment are related.

The aim of this study was to investigate the effects of alcohol intake and acute alcohol-related dysfunction on employment status using longitudinal data, in particular whether acute alcohol-related dysfunction was a mediator of the relationship between alcohol intake and employment. We used longitudinal data from the Izhevsk Family Studies carried out in Izhevsk, Russia, which collected detailed information on alcohol consumption, including several measures of acute dysfunctional behaviours.

## METHODS

### Study sample

The Izhevsk Family Study 1 (IFS-1) included a cross-sectional survey conducted between 2003 and 2006 of 1941 men aged 25–54 years selected from the 2002 population register of the city of Izhevsk. Most of these men (*n* = 1750) had been selected originally as live controls in a case–control study of the relationship between hazardous drinking and premature mortality [10], which involved them being frequency matched by age to cases (deceased men aged 25–54 years resident in Izhevsk). An additional 250 men identified from the same source were recruited in 2006. These were a random sample frequency matched by age to the distribution of the 1750 controls. For the majority of the men a proxy respondent living in the same household was also interviewed. Between 2008 and 2009 an attempt was made to locate these men and re-interview them as part of a follow-up study known as the Izhevsk Family Study 2 (IFS-2). For the purposes of this study, only the 1143 men who were in regular paid employment with a proxy interviewed at IFS-1 and who were also re-interviewed themselves at IFS-2 were included.

### Analytical strategy

The study was restricted to men who were in regular employment at baseline in order to clarify the potential temporal sequence of events (i.e. effects of alcohol use on employment status rather than effects of being unemployed on drinking).

### Outcome

The outcome of interest was self-reported employment status at the follow-up interview (IFS-2). Employment in Russia includes casual non-permanent jobs. Enforced unpaid leave and wage arrears (not being paid on time) are also common [11]. Job instability as well as unemployment have been shown to be associated with poorer health and mortality in Russia [11,12]. As regular paid employment is the most secure form of employment, the outcome of interest was defined as whether or not men were in regular paid employment at IFS-2. Men in irregular employment were included in the same category as men who were unemployed as ‘not in regular paid employment’ because, as discussed above, job instability as well as unemployment is associated with poorer health and the transition from regular paid employment at IFS-1 to irregular paid employment at IFS-2 is a negative change in employment status.

### Exposures

The exposures of interest were alcohol intake and acute alcohol-related dysfunction assessed at the baseline interview (IFS-1). All questions on alcohol use were asked with reference to the past 12 months.

#### Alcohol intake

The IFS-1 interview contained questions on the frequency of drinking beer, wine and spirits and the usual volume of each beverage consumed per drinking occasion asked in categories that are used in Russia in daily life (beer in bottles and wine and spirits in grams). These questions were used to calculate the total volume of ethanol from beverage alcohol consumed in litres per year. This variable was skewed to the right, therefore total volume of beverage alcohol was used either as a categorical variable or the log was used.

A characteristic feature of Russian drinking is the relatively high prevalence of consuming non-beverage alcohols [13]. These are sources of ethanol which are not intended for consumption, such as medicinal tinctures and eau de cologne [14]. Non-beverage alcohol consumption was used as a binary variable (yes/no).

#### Acute alcohol-related dysfunction

Alcohol-related dysfunction was divided into two types: routine dysfunction and sporadic dysfunction.

**Figure 1:**
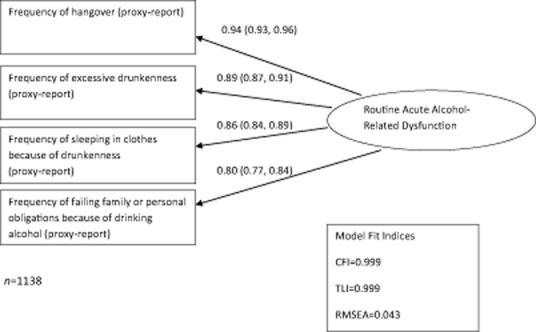
Measurement model for routine acute alcohol-related dysfunction at Izhevsk Family Study 1 (IFS-1) with standardized factor loadings (95% confidence intervals)

Routine dysfunction was measured using a latent variable manifested by observed frequency of four alcohol-related dysfunctions: hangover, excessive drunkenness, sleeping in clothes because of drunkenness and failing family or personal obligations because of drinking (Fig. 1). There were seven response categories for these questions: never or almost never, less than once a month, once a month, several times a month, once a week, several times a week and every day. Predicted scores on this underlying latent variable derived using data on all men (factor scores) were used to create an ordered categorical measure of dysfunction, with seven categories dividing men with any level of dysfunction into fifths of dysfunction score and separating non-drinkers and drinkers with no dysfunction (who both have a factor score equivalent to zero) using observed self-report of drinking status.

Sporadic dysfunction was measured by whether men had one or more episodes of *zapoi* in the past year. *Zapoi* is a feature of Russian drinking defined for participants as a period of continuous drunkenness of several days or more during which a person does not work and is withdrawn from normal life.

#### Self-versus proxy-reported data

All alcohol variables measured at IFS-1 were reported both by the man himself and by a proxy respondent living in the same household. Studies investigating agreement between proxy and self-report have generally found good levels of agreement for directly observable drinking behaviours, overall pattern of drinking and frequency of consumption, but less agreement for amount of ethanol consumed [15,16]. The same was found at IFS-1 [17]. Many of the questions used at IFS-1 were on drinking behaviours observable directly by a proxy. As proxy respondents would have less reason to under-report drinking patterns and dysfunctional behaviours (e.g. because of social desirability) it was considered that proxy response on these variables would be more accurate than self-report and therefore proxy reports of non-beverage alcohol consumption, *zapoi* and frequency of acute dysfunctional behaviours were used in the analyses. Self-reported data were used to calculate volume of ethanol, as it would be very difficult for a proxy to estimate accurately the volume consumed.

### Potential confounding variables

Variables considered as confounders were age, education, marital status, household amenities (access to a car or central heating), smoking status and having one or more chronic health problems (registered disabled and/or always had a cough in the morning in recent months and/or became breathless climbing stairs in recent months and/or had difficulty walking 1 km in recent months and/or difficulties in the activities of daily living such as washing or getting dressed in recent months). All these variables were assessed from self-reported data at baseline. Age, marital status, education and household amenities were considered as confounders, as they were likely to be associated independently with both alcohol use and employment status. Smoking status was considered a potential confounder, as alcohol use and smoking are associated strongly and smoking may lead independently to loss of employment through its effects on health. Health status was also considered as a confounder, as health status could affect both drinking and employment independently. However, as health problems may also be on the causal pathway between alcohol and employment, models are presented with and without adjustment for health status.

### Effect modifiers

A priori, we considered that there may be effect modification in the relationship between alcohol use and employment by type of occupation, as men working in certain occupations may be more (or less) vulnerable to the impact of alcohol on their work life. Occupation was classified into two groups: manual workers (skilled and unskilled workers) and non-manual workers (senior officials, managers, professionals, office clerks and entrepreneurs). A binary variable was used in order to maximize the power available to assess interactions. Education could also be a potential effect modifier; however, too few men with higher education became unemployed between the two surveys to assess this formally.

### Statistical analysis

Separate logistic regression models were fitted investigating the effects of each exposure variable on employment status sequentially adjusted for (i) age, (ii) other socio-demographic variables and smoking status and (iii) health problems. Models were not mutually adjusted for the other alcohol variables because these variables were all highly correlated. In the logistic regression model fifths of factors scores on routine acute alcohol-related dysfunction were included as categorical variables, but in addition the estimated effect of the continuous latent variable was obtained from a structural equation model specifying a direct effect of routine alcohol-related dysfunction on employment status, adjusting for confounders in the same way as in the logistic regression models.

Effect modification was assessed by entering interactions between drinking variables and occupation type in the fully adjusted logistic regression models.

**Figure 2:**
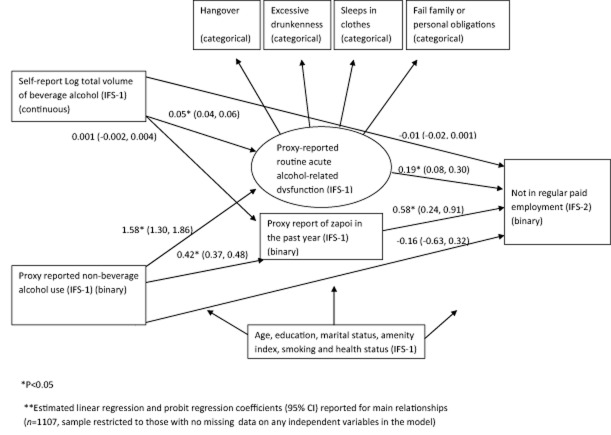
Structural equation model of the relationship between alcohol intake (volume of ethanol from beverage alcohol and non-beverage alcohol use), acute alcohol-related dysfunction (latent factor of acute dysfunction and *zapoi*) and employment status

The inter-relationship between alcohol intake and acute alcohol-related dysfunction at IFS-1 and employment status at IFS-2 was investigated by fitting the structural equation model shown in Fig. 2. This model was used to estimate both the direct effects of alcohol intake variables (log volume of ethanol and non-beverage alcohol use) at IFS-1 on employment status at IFS-2 and their indirect effects via acute alcohol-related dysfunction (latent factor of routine dysfunction and *zapoi*). Probit regression was used for these analyses in order to separate direct and indirect effects of the alcohol intake variables, because the outcome (employment status) was binary. These results depend upon the assumption that there are no unmeasured confounders for the exposure–outcome, exposure–mediator and mediator–outcome relations [18]. Sample size was considered adequate, as more than 10 cases per variable were included in the model [19]. For the structural equation models estimation was by weighted least squares with mean and variance adjusted (WLSMV). Model fit was assessed using the comparative fit index (CFI), the Tucker–Lewis Index (TLI) and the root mean square error of approximation (RMSEA). CFI and TLI values greater than 0.95 indicate good model fit, with a minimum of 0.90 indicating acceptable fit [19,20]. For the RMSEA, values greater than 0.10 indicates a bad fit, while less than 0.08 indicates a reasonable fit and values less than 0.05 indicate a good fit [20].

### Sensitivity analyses

1. The analyses for volume of beverage alcohol were repeated, excluding men if either they or their proxy reported non-beverage alcohol consumption or data on non-beverage alcohol use were missing. The purpose of this analysis was to determine if excluding non-beverage alcohol drinkers altered the results because including non-beverage alcohol drinkers may have resulted in bias, as men who drink non-beverage alcohol may drink comparatively low volumes of beverage alcohol but, in reality, drink a large volume of ethanol per year (which would not be measured by questions on consumption of beer, wine and spirits), and therefore including non-beverage alcohol drinkers may have obscured the true relationship between total volume of ethanol and unemployment.
2. The analyses for acute alcohol-related dysfunction were repeated, excluding men whose proxies reported that they had ‘serious work or employment-related problems’ at any time in the past 5 years. This was to investigate whether or not results were biased by proxies who perceived men as having problems at work being more likely to report drinking behaviour as dysfunctional.

### Missing data

Missing data due to item non-response at IFS-1 were dealt with in different ways for the two analysis methods. Logistic regression analyses were restricted to complete case analysis. For structural equation models, an analysis method equivalent to pairwise present analysis was used [21]. Both methods are valid under the assumption that missingness was completely at random.

Statistical analyses were carried out using Stata 12 [22] and Mplus 5 [21].

## RESULTS

There were 1619 of 1941 men in regular paid employment at IFS-1, of whom 1502 had proxy-reported data available at IFS-1. Of these men, 1143 (76.1%) were re-interviewed at IFS-2. There was good evidence that men who were included were more likely to be married (82.4 versus 75.0%, *P* = 0.007) and have less frequent hangovers by proxy report (*P* = 0.05), but no evidence for a difference in any other socio-demographic, health or drinking variables compared to all men in employment at baseline. The baseline characteristics of these 1143 men by employment status at IFS-2 are shown in Table 1. At IFS-2, 115 of 1143 men (10.1%) were no longer in regular paid employment. The percentage of men no longer in regular employment at IFS-2 was higher for older men and lower for those who were married, had higher education and owned both a car and central heating at IFS-1.

**Table 1:** Baseline characteristics of men in regular paid employment at Izhevsk Family Study 1 (IFS-1) by employment status at IFS-2

*Characteristic at IFS-1*		*n (%)*	*Number no longer in regular paid employment at IFS-2 (row %)*
Age (years)	25–29	77 (6.7)	10 (13.0)
	30–34	101 (8.8)	5 (5.0)
	35–39	104 (9.1)	8 (7.7)
	40–44	192 (16.8)	9 (4.7)
	45–50	272 (23.8)	24 (8.8)
	50–54	390 (34.1)	57 (14.6)
	55+	7 (0.6)	2 (28.6)
Marital status	Living with spouse in registered marriage	942 (82.4)	91 (9.7)
	Living with spouse not in registered marriage	109 (9.5)	14 (12.8)
	Divorced	36 (3.2)	4 (11.1)
	Widower	6 (0.5)	2 (33.3)
	Never married	50 (4.4)	4 (8.0)
Education	Incomplete secondary	56 (4.9)	9 (16.1)
	Secondary	820 (71.7)	92 (11.2)
	Higher	267 (23.4)	14 (5.2)
Amenity index	Neither car nor central heating	67 (5.9)	10 (14.9)
	Car or central heating	584 (51.1)	67 (11.5)
	Car and central heating	492 (43.0)	38 (7.7)
Smoking status (missing = 1)	Never smoked	227 (15.2)	22 (9.7)
	Ex-smoker	174 (35.1)	12 (6.9)
	Current smoker	741 (64.9)	81 (10.9)
Health problems^a^ (missing = 9)	No	646 (56.5)	57 (8.8)
	Yes	488 (42.7)	58 (11.9)
Occupation type (missing = 6)	Manual	745 (65.2)	87 (11.7)
	Non-manual	392 (34.3)	26 (6.6)
Total volume of ethanol from beverage alcohol in litres per year (missing = 14)	>0–2 litres	196 (17.4)	18 (9.2)
	2–4 litres	243 (21.5)	19 (7.8)
	5–9 litres	269 (23.8)	32 (11.9)
	10–19 litres	171 (15.2)	17 (9.9)
	20+ litres	104 (9.2)	12 (11.5)
Proxy report of drinking non-beverage alcohol (missing = 13)	Non-drinker	145 (12.7)	12 (8.3)
	Drinks beverage alcohol only	922 (81.6)	86 (9.3)
	Drinks non-beverage alcohol	63 (5.6)	15 (23.8)
Proxy report of *zapoi* in the past year	No (drinker)	924 (80.8)	83 (9.0)
	Yes (drinker)	74 (6.5)	20 (27.0)
Proxy report of hangover (missing = 35)	Never	670 (60.5)	50 (7.5)
	Less than once a month	218 (19.1)	18 (8.3)
	Once a month	114 (10.0)	20 (17.5)
	Several times a month	57 (5.0)	14 (24.6)
	Once a week	27 (2.4)	4 (14.8)
	Several times a week	16 (1.4)	0 (0.0)
	Every day	6 (0.5)	3 (50.0)
Proxy report of excessive drunkenness (missing = 17)	Never	618 (54.1)	49 (7.9)
	Less than once a month	255 (22.3)	19 (7.5)
	Once a month	131 (11.5)	25 (19.1)
	Several times a month	57 (5.0)	12 (21.1)
	Once a week	36 (3.1)	3 (8.3)
	Several times a week	20 (1.7)	3 (15.0)
	Every day	9 (0.8)	3 (33.3)
Proxy report of sleeping in clothes at night because of drunkenness (missing = 6)	Never	934 (81.7)	75 (8.0)
	Less than once a month	87 (7.6)	12 (13.8)
	Once a month	58 (5.1)	14 (24.1)
	Several times a month	23 (2.0)	5 (21.7)
	Once a week	13 (1.1)	2 (15.4)
	Several times a week	19 (1.7)	5 (26.3)
	Every day	3 (0.3)	2 (66.7)
Proxy report of failing family or personal obligations because of drinking (missing = 19)	Never	901 (78.8)	81 (9.0)
	Less than once a month	76 (6.6)	6 (7.9)
	Once a month	65 (5.7)	10 (15.4)
	Several times a month	39 (3.4)	7 (18.0)
	Once a week	25 (2.2)	4 (16.0)
	Several times a week	13 (1.1)	3 (23.1)
	Every day	5 (0.4)	2 (40.0)
Proxy report of acute alcohol-related dysfunction (latent) (missing = 5)^b^	Drinker: no dysfunction	386 (33.9)	28 (7.3)
	1st fifth of dysfunction	137 (12.0)	13 (9.5)
	2nd fifth of dysfunction	125 (11.0)	6 (4.8)
	3rd fifth of dysfunction	102 (9.0)	11 (10.8)
	4th fifth of dysfunction	128 (11.3)	23 (18.0)
	5th fifth of dysfunction	115 (10.1)	22 (19.1)
Total		1143 (100)	115 (10.1)

a Registered disabled and/or breathless climbing stairs and/or difficulty walking 1 km and/or always has a cough in the morning and/or problems with activities of daily living.

b Data missing for all four manifest variables: hangover, excessive drunkenness, sleeping in clothes because of drunkenness and failing family and personal obligations because of drinking.

The measurement model used to define acute alcohol-related dysfunction is shown in Fig. 1 with standardized factor loadings and model fit indices. All four manifest variables were associated strongly with the underlying latent factor and the model had good fit.

The prevalence of sporadic (*zapoi*) and routine dysfunction by the two measures of alcohol intake are shown in Table 2. There was strong evidence that both measures of alcohol intake were associated with both types of dysfunction; however, compared to the highest category of beverage alcohol consumption (greater than 20 litres of ethanol per year), non-beverage alcohol users had a higher prevalence of both *zapoi* (47.6 versus 18.3%) and routine dysfunction (76.2% with scores in the top two-fifths of dysfunction versus 62.5%).

**Table 2:** Prevalence of sporadic (*zapoi*) and routine alcohol-related dysfunction by alcohol intake at Izhevsk Family Study 1 (IFS-1) among drinkers

*Alcohol intake variables at IFS-1*		*Prevalence of proxy-reported routine acute alcohol-related dysfunction*^a^*(%)*	*Prevalence of proxy-reported zapoin (%)*
Volume of ethanol from beverage alcohol (litres per year) missing = 14	>0–2 litres	17/194 (8.8)	6/196 (3.1)
	2–4 litres	35/242 (14.5)	20/243 (8.2)
	5–9 litres	69/268 (25.7)	22/269 (8.2)
	10–19 litres	52/170 (30.6)	6/171 (3.5)
	20+ litres	65/104 (62.5)	19/104 (18.3)
	χ^2^ (df)	124.5 (4) *P* < 0.001	26.7 (4) *P* < 0.001
	Test for linear trend	*P* < 0.001	*P* = 0.002
Non-beverage alcohol drinker missing = 13^b^	No	188/921 (20.4)	42/922 (4.6)
	Yes	48/63 (76.2)	30/63 (47.6)
	χ^2^ (df)	100.6 (1) *P* < 0.001	164.6 (1) *P* < 0.001
Total^c^		242/993 (24.4)	74/998 (7.4)

a Defined as having a factor score on the latent variable in the top two-fifths of the sample. Missing for five men.

b There are four men missing data on both non-beverage alcohol consumption and routine alcohol-related dysfunction.

c There are 998 drinkers in the sample. One man reports drinking non-beverage alcohol only (drinker but volume of ethanol per year is zero).

The relationship between alcohol intake and employment is shown in the top half of Table 3.There was only very weak evidence for a positive association between volume of ethanol and employment. This remained the case when men who drank non-beverage alcohol were excluded (data not shown). In contrast, there was good evidence that drinkers who drank non-beverage alcohols were more likely to have ceased regular paid employment at IFS-2 compared to beverage-only drinkers even after adjusting for socio-demographic factors and health problems. There was no evidence of an interaction between occupation type and either volume of ethanol (*P* = 0.35) or non-beverage alcohol use (*P* = 0.42).

**Table 3:** Association between alcohol variables at Izhevsk Family Study 1 (IFS-1) and not being in regular paid employment at IFS-2

*Alcohol use at IFS-1* (N *=* 1143)		*Model 1*^a^,^f^		*Model 2*^c^,^f^		*Model 3*^c^,^f^	
		*Odds ratio (95%CI)*	*P-value*	*Odds ratio (95%CI)*	*P-value*	*Odds ratio (95%CI)*	*P-value*
Total volume of ethanol from beverage alcohol in litres per year (missing = 14)	Non-drinker	0.98 (0.46, 2.08)	Test for linear trend *P* = 0.19	0.94 (0.44, 2.02)	Test for linear trend *P* = 0.33	0.92 (0.43, 1.97)	Test for linear trend *P* = 0.36
	>0–2 litres	1.00 (ref)		1.00 (ref)		1.00 (ref)	
	2–4 litres	0.86 (0.44, 1.69)		0.88 (0.45, 1.75)		0.86 (0.44, 1.71)	
	5–9 litres	1.40 (0.76, 2.59)		1.40 (0.75, 2.60)		1.37 (0.74, 2.55)	
	10–19 litres	1.18 (0.59, 2.39)		1.10 (0.54, 2.24)		1.08 (0.53, 2.19)	
	20+ litres	1.43 (0.65, 3.10)		1.22 (0.55, 2.70)		1.17 (0.52, 2.61)	
	Log total volume of ethanol (continuous)	1.07 (0.96, 1.20)	Test for linear trend *P* = 0.22	1.06 (0.95, 1.19)	Test for linear trend *P* = 0.30	1.06 (0.95, 1.19)	Test for linear trend *P* = 0.32
Proxy report of non-beverage alcohol use (missing = 13)	Non-drinker	0.85 (0.45, 1.60)	Test for heterogeneity *P* = 0.006	0.83 (0.43, 1.57)	Test for heterogeneity *P* = 0.03	0.82 (0.43, 1.57)	Test for heterogeneity *P* = 0.04
	No	1.00 (ref)		1.00 (ref)		1.00 (ref)	
	Yes	2.88 (1.55, 5.38)		2.37 (1.24, 4.52)		2.30 (1.21, 4.40)	
Proxy report of *zapoi*	Non-drinker	0.89 (0.47, 1.67)	Test for heterogeneity *P* < 0.001	0.86 (0.45, 1.65)	Test for heterogeneity *P* = 0.001	0.86 (0.45, 1.64)	Test for heterogeneity*P* = 0.001
	No	1.00 (ref)		1.00 (ref)		1.00 (ref)	
	Yes	3.65 (2.08, 6.42)		3.10 (1.73, 5.53)		3.08 (1.71, 5.55)	
Fifths of proxy report of acute alcohol-related dysfunction (latent) (missing = 7**)**	Non-drinker^d^	1.15 (0.57, 2.33)	Test for linear trend *P* < 0.001	1.07 (0.52, 2.20)	Test for linear trend *P* < 0.001	1.07 (0.52, 2.19)	Test for linear trend *P* < 0.001
	Drinker: no dysfunction^d^	1.00 (ref)		1.00 (ref)		1.00 (ref)	
	First fifth of dysfunction	1.38 (0.69, 2.76)		1.28 (0.64, 2.58)		1.29 (0.64, 2.59)	
	Second fifth of dysfunction	0.70 (0.28, 1.72)		0.67 (0.27, 1.67)		0.66 (0.26, 1.65)	
	Third fifth of dysfunction	1.61 (0.77, 3.36)		1.50 (0.71, 3.17)		1.50 (0.71, 3.17)	
	Fourth fifth of dysfunction	2.89 (1.59, 5.25)		2.54 (1.38, 4.74)		2.57 (1.38, 4.78)	
	Fifth fifth of dysfunction	3.01 (1.65, 5.52)		2.77 (1.40, 4.95)		2.64 (1.40, 4.99)	
Proxy report of acute alcohol-related dysfunction (latent)^e^		1.60 (1.29, 1.99)	Test for linear trend *P* < 0.001	1.51 (1.21, 1.89)	Test for linear trend *P* < 0.001	1.50 (1.20, 1.88)	Test for linear trend *P* < 0.001

a Model 1: adjusted for age.

b Model 2: model 1 + education + marital status + level of amenities + smoking status.

c Model 3: model 2 + health problems.

d Both non-drinkers and drinkers with no dysfunction have a dysfunction score of zero but are distinguished here using the observed variable self-reported drinking status.

e Odds ratio refers to the increase in odds of no longer being employed at IFS-2 per standard deviation increase in the latent factor of acute alcohol-related dysfunction at IFS-1.

f Models are separate for each alcohol variable (i.e. not mutually adjusted for effects of the other alcohol variables). Sample size differs depending on amount of missing data on each alcohol variable.

The relationship between alcohol-related dysfunction and employment is shown in the bottom half of Table 3.

After adjusting for socio-demographic confounders (model 2) there was strong evidence that men who had been on *zapoi* in the previous year at IFS-1 had more than three times higher odds of having ceased regular paid employment at IFS-2. It was not possible to assess evidence of interaction between *zapoi* and occupation type, because all the non-manual workers who had experienced *zapoi* (*n* = 12) remained in regular paid employment at IFS-2.

After adjusting for confounders (model 2), there was strong evidence that drinkers in the top two-fifths of latent routine dysfunction had more than twice the odds of being unemployed at IFS-2 than drinkers with no dysfunction. When the latent factor of routine acute alcohol-related dysfunction was used as a continuous variable, the odds of no longer being in regular paid employment increased by 51% [95% confidence interval (CI) = 20–89%] for every standard deviation unit increase in dysfunction score. Additional adjustment for health problems (model 3) had very little impact on the association between alcohol-related dysfunction and employment, suggesting that the association between alcohol and employment was not mediated importantly through any negative effect on chronic health problems. There remained strong evidence of an association between dysfunction and employment status when men whose proxies reported ‘serious work-related or employment problems’ at IFS-1 were excluded (data not shown). There was no evidence of interaction between fifth of dysfunction score and occupation type (*P* = 0.21).

The relationships between alcohol intake (volume of ethanol and non-beverage alcohol use) and acute alcohol-related dysfunction (latent factor of routine alcohol-related dysfunction and *zapoi*) with employment are shown in Fig. 2 and Table 4. All results are shown with adjustment for health problems. Direct and indirect effects of non-beverage alcohol use and volume of ethanol on employment status are shown in Table 4. Non-beverage alcohol use had strong indirect effects on employment via both *zapoi* and routine acute alcohol-related dysfunction, but there was no evidence that non-beverage alcohol use had a direct effect on employment status once *zapoi* and routine alcohol-related dysfunction were included in the model. Volume of ethanol had no indirect effect via *zapoi*, but there was strong statistical evidence of a small indirect effect via routine alcohol-related dysfunction. There was strong statistical evidence that both *zapoi* and the latent factor of routine alcohol-related dysfunction directly influenced employment status at IFS-2.

**Table 4:** Acute alcohol-related dysfunction (*zapoi* and latent factor of routine alcohol-related dysfunction) as mediators of the relationship between alcohol intake (volume of ethanol from beverage alcohol and non-beverage alcohol use) at Izhevsk Family Study 1 (IFS-1) and employment at IFS-.2

*Alcohol variable at IFS-1 n* *=* 1107	*Employment at IFS-2*^a^					
	*Direct*		*Indirect via acute alcohol-relate dysfunction*		*Indirect via zapoi*	
	*Probit coefficient (95% CI)*	*P-value*	*Probit coefficient (95% CI)*	*P-value*	*Probit coefficient (95% CI)*	*P-value*
Self-reported log total volume of ethanol from beverage alcohol	−0.01 (−0.02, 0.002)	0.07	0.01 (0.002, 0.02)	0.002	0.001 (−0.001, 0.002)	0.54
Proxy-reported non-beverage alcohol use	−0.16 (−0.63, 0.32)	0.52	0.30 (0.11, 0.48)	0.002	0.25 (0.10, 0.39)	0.001
Proxy report of *zapoi*	0.58 (0.24, 0.91)	0.001	–		–	
Proxy-reported acute alcohol-related dysfunction (latent)	0.19 (0.08, 0.30)	0.001	–		–	
Model fit indices						
CFI	0.93					
TLI	0.89					
RMSEA	0.08					

a All models adjusted for age, education, marital status, level of amenities, smoking status and health problems at IFS-1. CI = confidence interval; CFI = comparative fit index; TLI = Tucker–Lewis Index (TLI); RMSEA = root mean square error of approximation.

## DISCUSSION

To our knowledge, this is the first study to consider the effects of acute alcohol-related dysfunction on employment status. We were able to assess this longitudinally and found that high levels of both sporadic (*zapoi*) and routine alcohol-related dysfunction at baseline were associated with higher odds of no longer being in regular paid employment at follow-up. Acute alcohol-related dysfunction was also an important mediator of the effects of alcohol intake (volume of ethanol from beverage alcohol and non-beverage alcohol use) on employment. Once alcohol-related dysfunction was included in the model there was no evidence that either measure of alcohol intake directly increased the probability of no longer being in regular employment. No evidence was found of interaction by occupation type.

The findings for total volume of beverage alcohol are in contrast to findings from previous studies [2,3], including analyses of the Russian Longitudinal Monitoring Survey (RLMS), which found that higher average daily consumption of alcohol increased the probability of job loss a year later [2]. There are some differences between the two studies: the RLMS asked questions on the frequency of consumption of all alcohol and usual daily consumption of beer, wine, spirits and home-made liquor (which was not measured at IFS-1) in the past 30 days. The authors used a measure of daily alcohol intake calculated from these data but do not explain how this was calculated. In addition, the outcome of interest was specifically whether men were fired, and therefore at follow-up only men who were no longer employed but still participating in the work-force were of interest (i.e. men who were in irregular employment or unemployed but not seeking work were not included as ‘unemployed’) and the period of follow-up was shorter. It is unclear if these differences would be sufficient to explain the discrepancy in the results of the two studies. It is worth noting that in our study any effects of total volume of ethanol were very small in comparison to the effects of *zapoi* and routine alcohol-related dysfunction. The RLMS study had a larger sample size (*n* = 4173) than IFS-2, but the effect size found for average daily consumption of alcohol was also very small (probit regression coefficient 0.003 increase in probability of being fired per 10 g of alcohol per week) [2].

All data on alcohol use were obtained from self-or proxy report and therefore subject to measurement error. Using proxy reports of drinking behaviour may be more accurate than self-reported data, as proxies have less reason to under-report socially unacceptable behaviours. However, proxy report is not reliable for certain aspects of alcohol use such as volume of ethanol consumed per occasion, and therefore could not be used for measuring alcohol intake. Self-reported alcohol intake is very likely to be affected by measurement error, because when asked about usual frequency and volume of consumption participants often report their mode rather than mean consumption, ignoring less frequent heavy drinking episodes [23]. Dysfunctional drinking behaviours such as hangover may be easier to report accurately than volume of alcohol consumed, especially for proxy respondents, and therefore results may partly reflect more accurate measurement of alcohol consumption. However, while ethanol must be consumed in order to experience acute alcohol-related dysfunction, experience of dysfunction is not entirely a function of the amount of ethanol consumed but represents interaction between a hazardous drinking pattern and individual-level susceptibility to the acute effects of alcohol. The strong association between alcohol-related dysfunction and employment, compared to the small effects of volume of ethanol which were mainly via dysfunction, seems to suggest that, when considering effects on employment, whether alcohol leads to dysfunctional behaviour is more important than the overall amount consumed. In this study the prevalence of both sporadic (*zapoi*) and routine dysfunction was higher in non-beverage alcohol drinkers compared to those in the highest category of beverage alcohol consumption (greater than 20 litres, of ethanol per year) which may explain why non-beverage alcohol consumption predicted employment status in the logistic regression model while volume of beverage alcohol did not. This is supported by the finding that the effects of non-beverage alcohol use were completely explained by alcohol-related dysfunction in the structural equation model.

In addition to the more general findings with respect to acute alcohol-related dysfunction, this is the first study to investigate the effects of two distinctive features of Russian drinking on employment: non-beverage alcohol consumption and *zapoi*. Both were associated strongly with transition out of regular paid employment. The findings of this study are particularly important given the high levels of hazardous drinking found in Russia [24,25].

There are some limitations in terms of generalizability of these findings. First, the need for a proxy respondent at baseline meant that men who were living alone were excluded. These men are likely to have been different to those included and therefore results are not applicable to all men in Izhvesk. The age distribution of our study population was also skewed towards older men. Furthermore, men who were lost to follow-up were less likely to have been married at baseline; however, with the exception of frequency of hangover, drinking behaviour at baseline was not associated with whether or not they were followed-up, suggesting that these men do not represent a heavier drinking population. Despite the longitudinal study design there may have been some problems with reverse causality, as men may start to drink more hazardously in response to work-place problems even though they are still in employment at that time. Nevertheless, there remained strong evidence of an association between alcohol-related dysfunction and employment status even when men who were perceived as having ‘serious work or employment related problems’ at baseline were excluded. There was no evidence of interaction between occupation type and alcohol use on employment status at IFS-2, although the relatively small number of men who became unemployed between the studies meant that in order to increase power in detecting interaction we used a binary categorization of occupational type, which may not have been sensitive enough at identifying occupational groups at particular risk. Very few men with higher education became unemployed and therefore it was not possible to investigate interaction by education. There may also have been other effect modifiers related to employment, such as income, which were not measured in these studies. Therefore, these results should be interpreted with caution as they may not apply equally to men of all occupational types or educational level. Although we adjusted for chronic health problems, the measure used was relatively simple and so may not have accounted for all the effects of chronic ill health. However, adjusting for health problems made very little difference to the estimated effects of alcohol intake and dysfunction on employment, suggesting that this was not an important pathway. Finally, this study assessed quantitatively the relationship between alcohol use at baseline and employment status at follow-up (3–5 years later), but qualitative work is needed alongside this to understand fully the role of alcohol in employment transitions.

In conclusion, non-beverage alcohol use and both sporadic and routine alcohol-related dysfunction were related prospectively to remaining in employment. Acute alcohol-related dysfunction was an important mediator of the relationship between alcohol intake and employment and should be considered in addition to conventional measures of alcohol consumption when investigating the impact of alcohol consumption on work. If further studies support our findings, dysfunctional behaviour could be used for identifying those who would benefit from interventions to reduce alcohol consumption. Reducing dysfunctional behaviour should be considered an important treatment aim for hazardous drinkers alongside reducing overall consumption.
